# Effect of a Topical Combination of Latanoprost and Pranoprofen on Intraocular Pressure and the Ocular Surface in Open-Angle Glaucoma Patients

**DOI:** 10.1155/2018/7474086

**Published:** 2018-12-13

**Authors:** Simin Zhu, Dabo Wang, Jing Han

**Affiliations:** Department of Ophthalmology, Affiliated Hospital of Qingdao University, Qingdao, China

## Abstract

**Objective:**

A prospective study was performed to observe the effects of nonsteroidal anti-inflammatory drug (NSAID) eyedrops on intraocular pressure (IOP) and the ocular surface in primary open-angle glaucoma (POAG) patients treated with 0.005% latanoprost eyedrops.

**Methods:**

Forty-eight subjects were randomized into two study groups (NSAID and control). Latanoprost was continued for 10 weeks in all subjects. At the end of week 4, pranoprofen was added in the NSAID group, and treatment lasted for 4 weeks, whereas patients in the control group were treated with latanoprost alone. IOP was measured in both groups every 2 weeks, and the changes in the ocular surface in the NSAID group were evaluated once a month.

**Results:**

Pranoprofen addition resulted in a decrease in IOP in the NSAID group compared to the control group (*p* < 0.01). After pranoprofen was discontinued, IOP significantly increased in the NSAID group (*p* < 0.01), remaining approximately at the same IOP as when they were being treated with latanoprost alone. During the same examination, no significant variations in IOP were found in the control group. Patients who were treated with latanoprost alone showed gradual improvements in ocular surface symptom scores and conjunctival hyperemia scores during the first four weeks of treatment (*p* < 0.01). When pranoprofen eyedrops were added, ocular surface symptom scores decreased (*p* < 0.01), but conjunctival hyperemia scores did not change significantly.

**Conclusions:**

For POAG patients treated with latanoprost, the combination of pranoprofen can not only significantly enhance the latanoprost-induced IOP-lowering effect but also relieve the uncomfortable ocular symptoms caused by latanoprost.

## 1. Introduction

Glaucoma is the second leading cause of blindness worldwide following cataracts. The main manifestation of glaucoma is irreversible damage to visual function, which eventually leads to loss of vision. Primary open-angle glaucoma (POAG) is a common clinical type of glaucoma. In POAG, IOP is the primary risk factor for the development and progression of glaucoma, and studies have shown that IOP reduction can slow/prevent progression of glaucoma [1]. Previous prospective, randomized, long-term studies have demonstrated the importance of IOP reduction in slowing the progression of disease. Unless there are contraindications, drug therapy is still the most common treatment for intraocular hypotension in POAG. Garway-Heath et al. conducted a randomized, multicenter study and confirmed that IOP-lowering drugs can preserve the field of vision in patients with open-angle glaucoma, which further established the importance of drugs for treating POAG [2]. Topical IOP-lowering drugs include prostaglandin (PG) analogs, *β*-receptor blockers, *α*-adrenoceptor blockers, *α*-adrenoceptor agonists, carbonic anhydrase inhibitors, and pilocarpine [3]. Many glaucoma treatment guidelines currently list PG analogs as a first-line treatment. Using latanoprost eyedrops once per night can reduce IOP in patients with POAG by 25–35%, but the current data suggest that IOP needs to be reduced by 30–50% to achieve better clinical improvement [4]. Obviously, it is difficult to use PG analogs alone to reduce IOP to meet the clinical needs for drug treatment for glaucoma.

In recent years, compound preparations have gradually been developed for glaucoma treatment. Although they can increase the IOP-lowering effect of single drug, they also increase the side effects. For example, the commonly used compound preparations latanoprost and timolol maleate eyedrops could increase the IOP-lowering effects, but the side effects of timolol were also increased. As a result, the application of compound preparations containing *β*-receptor blockers is restricted in patients with asthma, severe obstructive pulmonary disease, bradycardia, etc. [5]. Therefore, it is important to identify a safer method to increase IOP-lowering effects of PGs without increasing side effects in POAG patients being treated with these drugs.

NSAIDs are potent inhibitors of PG synthesis and are now widely used as clinical anti-inflammatory treatments. Pranoprofen has the tricyclic structure of propionic acid compounds and is a commonly used NSAID. The main anti-inflammatory mechanism of pranoprofen involves inhibition of cyclooxygenase (COX) activity and prevention of the transformation of arachidonic acid (AA) derivatives into endogenous PGs [6]. Studies have shown that PGs combined with NSAIDs can further reduce IOP [7–9]. One study showed that pranoprofen can effectively treat the inflammation associated with dry eye, especially mild to moderate dry eye [10]. Therefore, in order to find a more effective and safe way to reduce IOP, we conducted a prospective, randomized, and controlled study to observe the effect of the combination of 0.005% latanoprost eyedrops with 0.1% pranoprofen eyedrops in IOP. In addition, the efficacy of pranoprofen for alleviating the uncomfortable symptoms and signs associated with latanoprost use in POAG patients was studied.

## 2. Patients and Methods

Patients who were diagnosed with POAG were selected from the Department of Ophthalmology at the Affiliated Hospital of Qingdao University. The inclusion criteria were as follows: (1) POAG patients were diagnosed with POAG by ophthalmologic examinations such as slit lamp, visual field, retinal nerve fiber layer (RNFL), anterior chamber angle, and ophthalmoscopy evaluations; (2) local adult male or female patients ≥18 years old; (3) those who had not been treated or who had been treated with only one antiglaucoma drug that was discontinued before enrollment (*β*-blockers were discontinued for at least 21 days, adrenergic inhibitors for 14 days, and cholinergic agents and carbonic anhydrase inhibitors for 5 days) [11]; (4) patients with glaucomatous optic nerve head cupping (i.e., a vertical cup-disc ratio of at least 0.5) and/or with notching of the neuroretinal rim, which is characteristic for glaucoma; and (5) patients with a corneal thickness ranging between 500 mm and 540 mm.

The exclusion criteria were as follows: (1) history of angle-closure glaucoma or other types of glaucoma; (2) previous incisional ocular surgery in either eye with a surgical incision; (3) suspected or confirmed hypersensitivity to latanoprost eyedrops and/or pranoprofen eyedrops; (4) severe cardiovascular, liver, kidney diseases, or any compromising systemic diseases; (5) pregnant or lactating women; (6) ocular infection or inflammation, such as acute conjunctivitis, severe blepharitis, or keratitis, within 3 months of enrollment; (7) history of using artificial tears within 2 months of enrollment; (8) history of autoimmune diseases that cause ocular surface damage; (9) use of systemic or topical glucocorticoids or immunosuppressive agents that cause ocular surface damage; and (10) use of systemic PGs or NSAIDs 1 month before follow-up.

**Figure 1:**
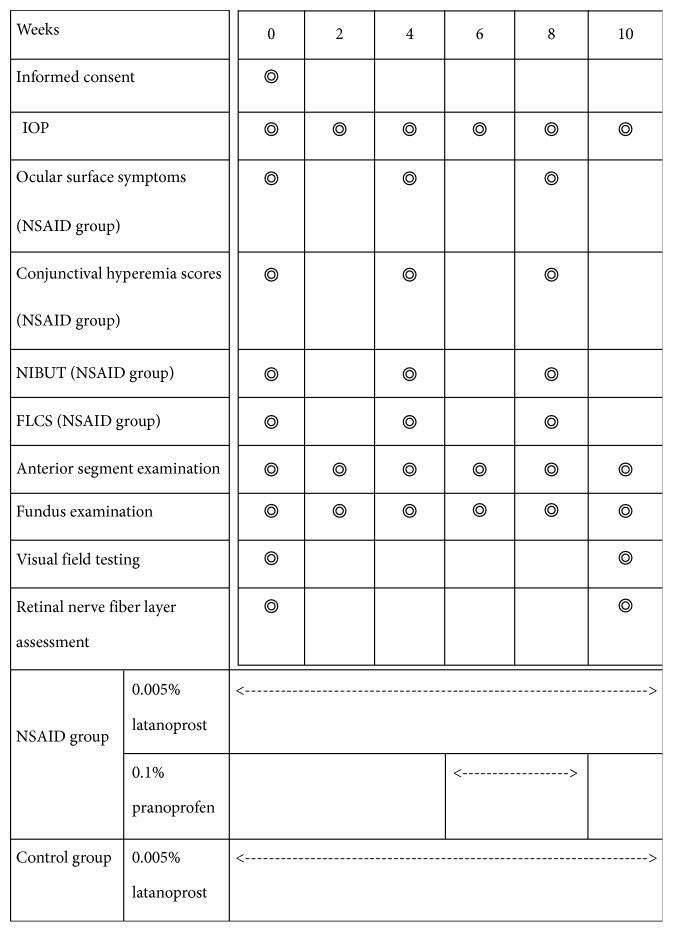
Processes performed during follow-up.

After the enrolled patients were informed of the study objectives and procedures, they signed an informed consent form to participate in the study. This study follows the principles of the Helsinki Declaration. A total of 48 eligible patients were randomized and then assigned to two different study groups (NSAID and control), according to the treatment protocols summarized in Figure 1.

In more specific detail, all patients continued topical treatment with 0.005% latanoprost (Pfizer, Belgium) once daily (at 9 pm) for 10 weeks. At the end of week 4, glaucoma patients in the NSAID group underwent 4 weeks of therapy with topical 0.1% pranoprofen eyedrops (Senju, Japan) three times daily (at 8 am, 12 noon, and 8 pm), whereas the patients in the control group were treated with latanoprost alone (at 9 pm). Both eyes were treated but only the index record from the right eye of each POAG patient was used for statistical analysis.

All IOP measurements were performed using a calibrated Goldmann applanation tonometer (AT 900 Mod.R) every 2 weeks, between 8 am and 10 am. Three fast IOP measurements were taken, and the values were averaged.

Changes in the ocular surface of the NSAID group were evaluated by ocular surface symptom scores and ocular surface signs (conjunctival hyperemia scores, noninvasive tear break-up time (NIBUT), and fluorescein staining) once a month during the same period (8 am–10 am) during follow-up.

Ocular surface symptom scores were assessed for the presence of four symptoms including dryness, foreign body sensation, tingling sensation, and itching sensation. The severity of the ocular surface symptoms was assessed on a 4-point scale from 0 to 3. Higher scores indicated worse clinical symptoms.

Conjunctival hyperemia scores included bulbar conjunctival hyperemia scores and palpebral conjunctival hyperemia scores. According to different clinical signs in the patients, the score ranged from 0 to 3 points. Higher scores indicated worse clinical signs.

NIBUT was assessed with a dry eye testing device (Oculus Keratograph, Germany). NIBUT was measured three times for each assessment, and the values were averaged.

Fluorescein staining (FLCS) was evaluated by applying fluorescein strips to the inferior fornix of the eye after moistening the strip with saline solution. If the coloration of the patient's corneal epithelium converged, 3 points were assigned; if the corneal epithelium was spotted, and the number of points was >5 without confluence, 2 points were assigned; if the corneal epithelium was spotted, and the number of points was ≤5 without confluence, 1 point was assigned; and if no coloration was present, 0 points were assigned.

The anterior segment and fundus were evaluated and monitored every two weeks by the same person. At the beginning of the study (week 0) and the end of week 10, an Octopus perimeter (Swiss Octopus 900 Pro) was used to detect visual field. Spectral-domain optical coherence tomography (OCT) (Spectralis OCT) was used to assess the RNFL.

All data were statistically analyzed with SPSS 22.0 software. The IOP measurements were compared between the two groups with two-way ANOVA (repeated measures). One-way ANOVA (repeated measures) was used for intragroup comparisons. Values of *p* < 0.05 were considered significant.

## 3. Results

**Figure 2:**
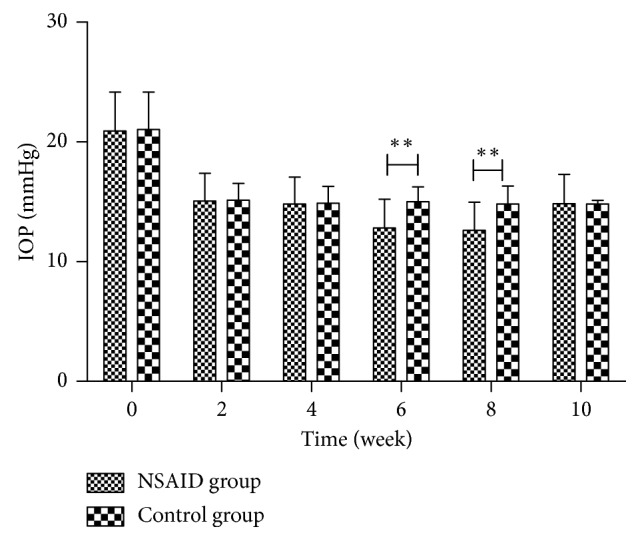
IOP differences between the two groups. ^*∗∗*^Intergroup comparison at the same time point with *p* < 0.05.

There were no significant differences in age, sex, or baseline IOP between the two study groups (Table 1). In both groups, administration of topical 0.005% latanoprost induced a significant reduction in IOP (NSAID group, week 0: 20.92 ± 3.25 mmHg versus week 2: 15.06 ± 2.34 mmHg; *p* < 0.01 and control group, week 0: 21.04 ± 3.11 mmHg versus week 2: 15.15 ± 1.40 mmHg; *p* < 0.01). In week 4, the IOP further decreased in both groups (NSAID group, week 2: 15.06 ± 2.34 mmHg versus week 4: 14.82 ± 2.25 mmHg; *p* < 0.05 and control group, week 2: 15.15 ± 1.40 mmHg versus week 4: 14.88 ± 1.41 mmHg; *p* < 0.05). The difference in IOP between the two groups was not statistically significant at week 2 and week 4 (week 2 comparison; *p* > 0.05 and week 4 comparison; *p* > 0.05). After pranoprofen was added in the NSAID group, patients exhibited a marked decrease in IOP (week 6: 12.81 ± 2.39 mmHg versus week 4: 14.82 ± 2.25 mmHg; *p* < 0.01), whereas there was no noticeable difference in IOP between week 4 and week 6 in the patients in the control group (week 6: 15.01 ± 1.22 mmHg versus week 4: 14.88 ± 1.41 mmHg; *p* > 0.05). The examinations performed during week 8 showed a further decrease in the IOP in the NSAID group (week 6: 12.81 ± 2.39 mmHg versus week 8: 12.60 ± 2.36 mmHg; *p* < 0.05). However, no significant difference in IOP was observed in the control group during week 8 (week 6: 15.01 ± 1.22 mmHg versus week 8: 14.82 ± 1.49 mmHg; *p* > 0.05). The difference in IOP between the two groups at this time point was statistically significant (week 6 comparison; *p* < 0.01 and week 8 comparison; *p* < 0.01). After pranoprofen was discontinued in the NSAID group, IOP significantly increased (week 10: 14.83 ± 2.47 mmHg versus week 8: 12.60 ± 2.36 mmHg; *p* < 0.01) approximately to the same IOP as when the patients were being treated with 0.005% latanoprost eyedrops alone (week 10: 14.83 ± 2.47 mmHg versus week 2: 15.06 ± 2.34 mmHg; *p* > 0.05 and week 10: 14.83 ± 2.47 mmHg versus week 4: 14.82 ± 2.25 mmHg; *p* > 0.05). In the control group, there were no significant differences in the mean IOP measurements at the same time points (week 10: 14.80 ± 1.47 mmHg versus week 8: 14.82 ± 1.49 mmHg; *p* > 0.05). Moreover, the IOP measurements recorded at the last examination were similar in both groups (week 10 comparison; *p* > 0.05) (Table 2; Figure 2).

**Figure 3:**
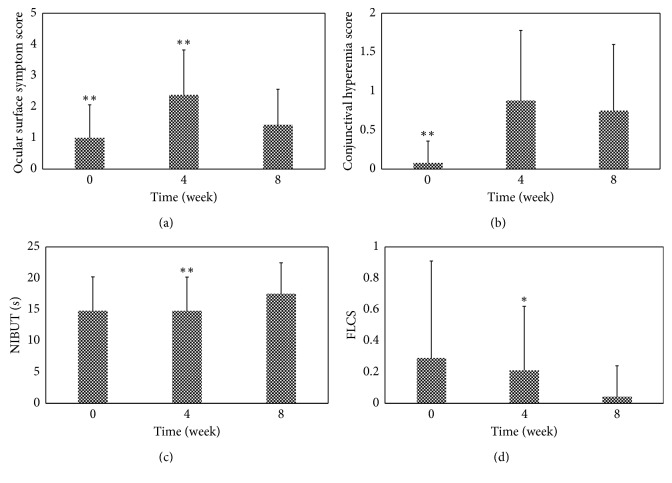
Changes in each ocular surface assessment parameter in the NSAID group. ^∗∗^Intragroup comparison between subsequent time points with *p* < 0.01; ^∗^intragroup comparison between subsequent time points with *p* < 0.05.

Analysis of the mean changes from baseline in ocular surface symptom scores and conjunctival hyperemia scores revealed progressive increases when patients were treated with 0.005% latanoprost alone for one month (ocular surface symptom scores, week 0: 1.00 ± 1.06 versus week 4: 2.38 ± 1.44; conjunctival hyperemia scores, week 0: 0.08 ± 0.28 versus week 4: 0.88 ± 0.90; *p* < 0.01). However, no significant differences in NIBUT and FLCS were observed during the same period (NIBUT, week 0: 14.79 ± 5.43 s versus week 4: 14.77 ± 5.41 s; FLCS, week 0: 0.29 ± 0.62 versus week 4: 0.21 ± 0.41; *p* > 0.05). Furthermore, after 4 weeks of treatment with 0.1% pranoprofen, both the ocular surface symptom scores and FLCS results had significantly decreased, and the NIBUT had increased (ocular surface symptom scores, week 8: 1.42 ± 1.14 versus week 4; *p* < 0.01; FLCS, week 8: 0.04 ± 0.20 versus week 4; *p* < 0.05; NIBUT, week 8: 17.52 ± 4.94 versus week 4; *p* < 0.01). However, no simultaneous significant difference in conjunctival hyperemia scores was observed (week 8: 0.75 ± 0.85 versus week 4; *p* > 0.05) (Table 3; Figure 3).

There were no significant changes in the anterior segment, fundus, visual field, or RNFL of the evaluated eyes during the follow-up period.

## 4. Discussion

Studies have shown that the main mechanism of action of PG derivatives in the treatment of glaucoma is reducing IOP by increasing the outflow of aqueous humor from the uveoscleral membrane [12]. However, their precise mechanism of action in human eyes remains to be studied. PGD2, PGE2, PGF2*α*, PGI2, and TXA2 are produced by AA and are combined with nine PG receptors (DP1, DP2, EP1, EP2, EP3, EP4, FP, IP, and TP). When PG derivatives bind to FP and TP receptors, IOP decreases. When they bind to DP, EP1–EP4, and IP receptors, IOP increases [13]. Each PG receptor binds preferentially to specific PGs, and PGs bind to all these receptors only when the PG concentration is too high. Therefore, it seems unrealistic to enhance the IOP-lowering effect of PGs by increasing the drug concentration. Latanoprost is a kind of PG derivative, PGF2*α*, and is an FP receptor agonist. Latanoprost mainly binds to FP, causing relaxation of the ciliary muscle and changes in the extracellular matrix of the ciliary muscle to cause aqueous humor outflow, resulting in an IOP-lowering effect [14].

Hardy and Abran et al. have reported that FP receptor density and binding strength are regulated by COX in neonatal and adult porcine retinal vessels. When COX is inhibited, both FP receptor density and binding strength increase in retinal vessels. It has also been observed that the FP receptor density is negatively correlated with PGF2*α* concentration [15, 16]. Can NSAIDs increase the FP receptor density and/or binding strength in the anterior segment of the eye by inhibiting the activity of COX, thereby enhancing the latanoprost-induced IOP-lowering effect? Costagliola et al. compared the effects of topical diclofenac sodium eyedrops on the IOP-lowering effect of latanoprost and timolol [7]. The NSAID diclofenac sodium can enhance the IOP-lowering effect of latanoprost, but no significant differences in IOP were observed in patients treated with timolol. Turanvural et al. also demonstrated that oral or topical application of the NSAID ketoproic acid can enhance the latanoprost-induced IOP-lowering effect [9]. We performed a prospective, randomized, controlled clinical study of 48 patients with POAG to observe the effects of combined treatment with pranoprofen and latanoprost followed by discontinuation of pranoprofen on the latanoprost-induced IOP-lowering effect. In the NSAID group, after administration of the combination of pranoprofen and latanoprost, IOP was further reduced, and there were no significant differences in IOP in the control group. The latanoprost-induced IOP-lowering effect increased from 28.64% to 39.54% when pranoprofen was added. However, after pranoprofen was discontinued two weeks later, the IOP-lowering effect returned to 28.57%. In the control group, the latanoprost-induced IOP-lowering effect fluctuated between 27.08 and 28.90%. Obviously, this indicated that pranoprofen and latanoprost have a synergistic IOP-lowering effect, and the synergistic effect completely disappeared after pranoprofen eyedrops were discontinued.

However, the results of studies by Chiba et al. [17], Kashinwagi and Tsukahara [18], and Taniguchi et al. [19] were different from ours. Their results showed that the combination of NSAIDs reduced the IOP-lowering effects of PGs. The reasons for these contradicting results may be related to race, the type of NSAIDs, and the subjects. Hedman and Larsson compared the efficacy of latanoprost eyedrops among patients with POAG and/or ocular hypertension (OH) in the United States, Asia, the Caucasus, and Mexico and showed that latanoprost was more effective in Asian and Mexican patients than American [20]. Therefore, race may be an important reason for the aforementioned contradictory results. Moreover, by comparison, we found that, in the studies by Costagliola et al. and Turanvural et al., they used diclofenac ophthalmic solution and ketorolac, respectively [7, 9], while in the studies by Chiba et al. and Kashiwagi and Tsukahara, bromfenac sodium was used [17, 18]; thus, the type of NSAIDs may have affected the results of the study. The members enrolled in Kashiwagi and Tsukahara's study were healthy subjects; however, the subjects in our trial were all patients with POAG. Previous studies have shown that the latanoprost-induced IOP-lowering effect is not as potent in healthy people as in patients with glaucoma. This may be related to the pathological upregulation of PG receptors in the iris and ciliary body in glaucoma patients [21]. Therefore, the inclusion of different subjects can also lead to the differences in results.

In this study, few patients had large differences in responses to the combination of pranoprofen and latanoprost for reducing IOP, which, in our opinion, may be related to their gender, age, individual differences, initial IOP, or other factors. Of course, there are limitations, such as the limited number of subjects and short observation time, in our study. In the future, a multicenter study should be carried out to further confirm whether the NSAID pranoprofen can enhance the latanoprost-induced IOP-lowering effect.

Studies have shown that long-term use of PG eyedrops causes a certain degree of damage to meibomian gland function and corneal structure and affect the health of the ocular surface [22]. We compared ocular surface symptom scores and conjunctival hyperemia scores between 0 and 4 weeks in the NSAID group and found that the short-term (one month) use of latanoprost eyedrops only affected ocular surface symptoms and caused conjunctival hyperemia, both which do not affect the stability of the tear film or damage the corneal epithelium. A study by W.G. El Hajj Moussa et al. also showed that the short-term use of latanoprost eyedrops caused ocular surface discomfort in patients [23]. However, Nie et al. showed that the continuous use of latanoprost eyedrops for 1 week, 1 month, 3 months, and even 1 year had no significant effect on NIBUT or corneal fluorescence staining [24], which is consistent with the results of our trial. At present, it is believed that the changes in ocular surface symptoms and conjunctival hyperemia caused by latanoprost are related to the following two factors: (1) 0.005% latanoprost eyedrops contain benzalkonium chloride (BKC), a preservative, which can cause changes in cell membrane permeability eventually, leading to dry eye and ocular surface inflammation [25, 26]; (2) binding of PGs to the PGE2 receptor may precipitate these changes. The PGE2 receptor has four subtypes: EP1, EP2, EP3, and EP4. PGE2 is widely present in the human body. During the development of inflammation, PGE2 participates in inflammation through its four receptors during different stages of inflammation. PGE2 also regulates and participates in both anti-inflammatory and proinflammatory states [27, 28]. Low concentrations of PGs preferentially bind to specific PG receptors, but high concentrations of PGs can bind to all receptors. The exogenous pharmacological preparation of 0.005% latanoprost eyedrops has a higher concentrations of PGs than of endogenous PGs, so they can bind with the PGE2 receptor and then increase the activity of COX to synthetize endogenous PGs, ultimately promoting the inflammatory reaction. Local vasodilation and increases in capillary permeability caused by the inflammatory reaction lead to inflammatory symptoms and signs, such as redness, swelling, heat, and pain.

Because of the few adverse reactions associated with pranoprofen eyedrops, which disappear automatically after drug withdrawal, these eyedrops have been widely used in clinical practice. Pranoprofen eyedrops not only inhibit the activity of COX and block the conversion of AA derivatives to endogenous PGs, thereby reducing inflammatory reactions, but also inhibit bradykinin, histamine, protein kinases, tumor necrosis factors, and other cytokines and activate anti-H1 receptors to produce anti-inflammatory effects. Chen et al. reported that pranoprofen can effectively control ocular surface inflammation in dry eye and has a favorable effect on the ocular surface [29]. In our study, we observed ocular surface symptoms and signs before and after administration of the combination of the two drugs in the NSAID group. The final results showed that after 4 weeks of treatment with these two drugs, the ocular surface symptom scores and FLCS results in week 8 were significantly lower than those in week 4, and the NIBUT was significantly increased, but the conjunctival hyperemia score did not change significantly. In our study, combination treatment with pranoprofen did not cause damage to the ocular surface but did relieve the uncomfortable ocular symptoms caused by PGs. Although pranoprofen can improve ocular surface inflammation in patients with dry eye, it does not improve the signs of conjunctival hyperemia caused by PGs. We speculate that this may be because pranoprofen can only alleviate ocular surface inflammation caused by BKC but has little influence on the inflammation caused by latanoprost itself.

In summary, it appears that combining pranoprofen with latanoprost in patients with POAG not only increases the latanoprost-induced IOP-lowering effect but also relieves the uncomfortable ocular symptoms caused by latanoprost. However, the diurnal IOP-lowering effect of the combination was not evaluated. Thus, for patients with POAG, further clinical observation studies are needed to determine whether this combination can stabilize IOP for a prolonged time period to delay or even prevent damage to the optic nerve caused by glaucoma, as well as to evaluate the risks and benefits of this treatment combination. The specific mechanism by which NSAIDs enhance the efficacy of PGs for reducing IOP remains to be further verified.

## Figures and Tables

**Table 1:** Study population characteristics.

	NSAID group	Control group
Total patients	24	24
Age range (years)	32.4 ± 4.7	29.6 ± 5.2
Gender (female/male)	13/11	10/14
Mean baseline IOP (mmHg)	20.92 ± 3.25	21.05 ± 3.11

**Table 2:** IOP in the two groups at different timepoints during the follow-up period.

Time (week)	IOP (mmHg, mean ± SD)	
	NSAID group	Control group
0	20.92 ± 3.25	21.04 ± 3.11
2	15.06 ± 2.34^*∗∗*^	15.15 ± 1.40^*∗∗*^
4	14.82 ± 2.25^*∗∗*^	14.88 ± 1.41^*∗∗*^
6	12.81 ± 2.39^*∗*^^,#^	15.01 ± 1.22
8	12.60 ± 2.36^*∗*^^,#^	14.82 ± 1.49
10	14.83 ± 2.47	14.80 ± 1.47

^#^Intergroup comparison at the same time point with *p* < 0.05; ^∗∗^intragroup comparison to week 0 with *p* < 0.05; ^∗^intragroup comparison to week 4 with *p* < 0.05.

**Table 3:** Mean result of each ocular surface assessment parameter in the NSAID group.

Time (week)	Ocular surface symptom score	Conjunctival hyperemia score	NIBUT (s)	FLCS
0	1.00 ± 1.06^##^	0.08 ± 0.28^##^	14.79 ± 5.43	0.29 ± 0.62
4	2.38 ± 1.44^##^	0.88 ± 0.90	14.77 ± 5.41^##^	0.21 ± 0.41^#^
8	1.42 ± 1.14	0.75 ± 0.85	17.52 ± 4.94	0.04 ± 0.20

^##^Intragroup comparison between subsequent time points with *p* < 0.01; ^#^intragroup comparison between subsequent examinations with *p* < 0.05.

## Data Availability

The primary data used to support the findings of this study can be obtained by contacting the first author through email (siminzhu1993@163.com).

## References

[1] Kim J. H., Caprioli J. (2018). Intraocular pressure fluctuation: is it important?. *Journal of Ophthalmic and Vision Research*.

[2] Garway-Heath D. F., Crabb D. P., Bunce C. (2015). Latanoprost for open-angle glaucoma (UKGTS): a randomised, multicentre, placebo-controlled trial. *The Lancet*.

[3] Harasymowycz P., Birt C., Gooi P. (2016). Medical management of glaucoma in the 21st century from a canadian perspective. *Journal of Ophthalmology*.

[4] Sihota R., Angmo D., Ramaswamy D., Dada T. (2018). Simplifying “target” intraocular pressure for different stages of primary open-angle glaucoma and primary angle-closure glaucoma. *Indian Journal of Ophthalmology*.

[5] Bilewicz-Stebel M. B., Miziołek B., Bergler-Czop B., Stańkowska A. (2018). Drug-induced subacute cutaneous lupus erythematosus caused by a topical beta blocker - timolol. *Acta Dermatovenerologica Croatica*.

[6] Schalnus R. (2003). Topical nonsteroidal anti-inflammatory therapy in ophthalmology. *Ophthalmologica*.

[7] Costagliola C., Parmeggiani F., Antinozzi P. P., Caccavale A., Cotticelli L., Sebastiani A. (2005). The influence of diclofenac ophthalmic solution on the intraocular pressure-lowering effect of topical 0.5% timolol and 0.005% latanoprost in primary open-angle glaucoma patients. *Experimental Eye Research*.

[8] Sponsel W. E., Paris G., Trigo Y. (2002). Latanoprost and brimonidine: therapeutic and physiologic assessment before and after oral nonsteroidal anti-inflammatory therapy. *American Journal of Ophthalmology*.

[9] Turanvural E., Torunacar B., Acar S. (2012). Effect of ketorolac add-on treatment on intra-ocular pressure in glaucoma patients receiving prostaglandin analogues. *Ophthalmologica*.

[10] He H., Liu Z. G., Lin Z. R., Liu X. C., He H., Xiao Q. G. (2012). Therapeutic effects of Pyranoprofen on the mouse dry eye induced by topical medication of benzalkonium chloride. *Chinese Journal of Ophthalmology*.

[11] Samuelson T. W., Katz L. J., Wells J. M., Duh Y.-J., Giamporcaro J. E. (2011). Randomized evaluation of the trabecular micro-bypass stent with phacoemulsification in patients with glaucoma and cataract. *Ophthalmology*.

[12] Aptel F., Denis P. (2011). Balancing efficacy and tolerability of prostaglandin analogues and prostaglandin–timolol fixed combinations in primary open-angle glaucoma. *Current Medical Research and Opinion*.

[13] Sharif N. A., Crider J. Y., Husain S., Kaddour-Djebbar I., Ansari H. R., Abdel-Latif A. A. (2003). Human ciliary muscle cell responses to FP-class prostaglandin analogs: phosphoinositide hydrolysis, intracellular Ca^2+^ mobilization and MAP kinase activation. *Journal of Ocular Pharmacology and Therapeutics*.

[14] Lim K. S., Nau C. B., O’Byrne M. M. (2008). Mechanism of action of bimatoprost, latanoprost, and travoprost in healthy subjects. A crossover study. *Ophthalmology*.

[15] Hardy P., Bhattacharya M., Abran D. (1998). Increases in retinovascular prostaglandin receptor functions by cyclooxygenase-1 and -2 inhibition. *Investigative Ophthalmology and Visual Science*.

[16] Abran D., Hardy P., Varma D. R., Chemtob S. (1995). Mechanisms of the biphasic effects of peroxides on the retinal vasculature of newborn and adult pigs. *Experimental Eye Research*.

[17] Chiba T., Kashiwagi K., Chiba N., Tsukahara S. (2006). Effect of non-steroidal anti-inflammatory ophthalmic solution on intraocular pressure reduction by latanoprost in patients with primary open angle glaucoma or ocular hypertension. *British Journal of Ophthalmology*.

[18] Kashiwagi K., Tsukahara S. (2003). Effect of non-steroidal anti-inflammatory ophthalmic solution on intraocular pressure reduction by latanoprost. *British Journal of Ophthalmology*.

[19] Taniguchi T., Haque M. S., Sugiyama K., Hori N., Kitazawa Y. (1996). Ocular hypotensive mechanism of topical isopropyl unoprostone, a novel prostaglandin metabolite-related drug, in rabbits. *Journal of Ocular Pharmacology and Therapeutics*.

[20] Hedman K., Larsson L. I. (2002). The effect of latanoprost compared with timolol in African-American, Asian, Caucasian, and Mexican open-angle glaucoma or ocular hypertensive patients. *Survey of Ophthalmology*.

[21] Abran D., Dumont I., Hardy P. (1997). Characterization and regulation of prostaglandin E2 receptor and receptor-coupled functions in the choroidal vasculature of the pig during development. *Circulation Research*.

[22] Schwartz G. F., Kotak S., Mardekian J., Fain J. M. (2011). Incidence of new coding for dry eye and ocular infection in open-angle glaucoma and ocular hypertension patients treated with prostaglandin analogs: retrospective analysis of three medical/pharmacy claims databases. *BMC Ophthalmology*.

[23] El Hajj Moussa W. G., Farhat R. G., Nehme J. C. (2018). Comparison of efficacy and ocular surface disease index score between bimatoprost, latanoprost, travoprost, and tafluprost in glaucoma patients. *Journal of Ophthalmology*.

[24] Nie L., Zhao Y., Junhua L. I. (2017). Effect of long-term topical anti-glaucoma medications on meibomian glands and tear film. *Journal of Wenzhou Medical University*.

[25] Panos G. D., Konstantinidis A., Mendrinos E., Kozobolis V., Perente I., Gatzioufas Z. (2013). Effect of tafluprost 0.0015% on central corneal thickness in patients with primary open-angle glaucoma. *Current Eye Research*.

[26] Lee A. J., Mccluskey P. (2010). Clinical utility and differential effects of prostaglandin analogs in the management of raised intraocular pressure and ocular hypertension. *Clinical Ophthalmology*.

[27] Biswas S., Bhattacherjee P., Paterson C. A., Tilley S. L., Koller B. H. (2006). Ocular inflammatory responses in the EP2 and EP4 receptor knockout mice. *Ocular Immunology and Inflammation*.

[28] Yuhki K., Ueno A., Naraba H. (2004). Prostaglandin receptors EP2, EP3, and IP mediate exudate formation in carrageenin-induced mouse pleurisy. *Journal of Pharmacology and Experimental Therapeutics*.

[29] Chen J., Dong F., Chen W. (2014). Clinical efficacy of 0.1% pranoprofen in treatment of dry eye patients: a multicenter, randomized, controlled clinical trial. *National Medical Journal of China*.

